# Binding of CXCL8/IL-8 to *Mycobacterium tuberculosis* Modulates the Innate Immune Response

**DOI:** 10.1155/2015/124762

**Published:** 2015-08-02

**Authors:** Agnieszka Krupa, Marek Fol, Bozena R. Dziadek, Ewa Kepka, Dominika Wojciechowska, Anna Brzostek, Agnieszka Torzewska, Jaroslaw Dziadek, Robert P. Baughman, David Griffith, Anna K. Kurdowska

**Affiliations:** ^1^Department of Cellular and Molecular Biology, University of Texas Health Science Center at Tyler, Tyler, TX 75708, USA; ^2^Institute of Medical Biology, Polish Academy of Sciences, 93-232 Lodz, Poland; ^3^Department of Immunology and Infectious Biology, University of Lodz, 90-237 Lodz, Poland; ^4^Department of Immunoparasitology, University of Lodz, 90-237 Lodz, Poland; ^5^Department of Immunobiology of Bacteria, University of Lodz, 90-237 Lodz, Poland; ^6^Department of Medicine, University of Cincinnati Medical Center, Cincinnati, OH 45219, USA; ^7^Department of Medicine, University of Texas Health Science Center at Tyler, Tyler, TX 75708, USA

## Abstract

Interleukin-8 (IL-8) has been implicated in the pathogenesis of several human respiratory diseases, including tuberculosis (TB). Importantly and in direct relevance to the objectives of this report quite a few findings suggest that the presence of IL-8 may be beneficial for the host. IL-8 may aid with mounting an adequate response during infection with *Mycobacterium tuberculosis (M. tb)*; however, the underlying mechanism remains largely unknown. The major goal of our study was to investigate the contribution of IL-8 to the inflammatory processes that are typically elicited in patients with TB. We have shown for the first time that IL-8 can directly bind to tubercle bacilli. We have also demonstrated that association of IL-8 with *M. tb* molecules leads to the augmentation of the ability of leukocytes (neutrophils and macrophages) to phagocyte and kill these bacilli. In addition, we have shown that significant amount of IL-8 present in the blood of TB patients associates with erythrocytes. Finally, we have noted that IL-8 is the major chemokine responsible for recruiting T lymphocytes (CD3^+^, CD4^+^, and CD8^+^ T cells). In summary, our data suggest that the association of IL-8 with *M. tb* molecules may modify and possibly enhance the innate immune response in patients with TB.

## 1. Introduction

*Mycobacterium tuberculosis* (*M. tb*) is an infectious agent that claims about three million lives each year [1]. Pathological manifestations of tuberculosis (TB) result from disregulated inflammatory responses. In contrast to adaptive (acquired) immunity, developed after contact with an antigen and mediated by T lymphocytes, the innate immune system is not dependent on memory of a previous exposure and therefore has no specificity. Thus, neutrophils, monocytes, macrophages, and natural killer cells constitute cellular effectors of innate immunity. Impairment of the functional integrity of this pivotal arm of the immune system leads to an increase in host susceptibility to infection with* M. tb* [2].

Interleukin-8 (IL-8) displays two major biological activities: chemoattraction and activation of several types of white blood cells. These properties of IL-8 can have important clinical consequences by affecting the pathogenesis of severe infectious diseases, including mycobacterial infections such as TB. IL-8 plays a central role in normal immune response to* M. tb* and has been shown to be absolutely required for granuloma formation [3]. Monocytes and macrophages infected with* M. tb* may be primary producers of IL-8 during the course of TB [4–6]; however, neutrophils as well as respiratory epithelial cells also have the ability to secrete this chemokine [3, 7, 8]. Moreover, IL-8 is most likely responsible for bringing neutrophils to sites of infection in patients with TB; for example, bronchoalveolar (BAL) fluids from these patients show a dramatic increase in neutrophil numbers which correlates with elevated concentrations of IL-8 [9, 10]. Similarly, the extent of expression of IL-8 mRNA in tuberculous lymph nodes is proportional to neutrophil infiltration [11]. The consequences of high levels of IL-8 secretion during pulmonary tuberculosis include the accumulation of neutrophils and the recruitment of T lymphocytes and monocytes [3, 12]. In addition, plasma IL-8 concentrations are higher in patients who died from TB than in survivors [9, 10]. On the other hand, IL-8 is required for effective host defense against* M. tb*. For instance, because of its angiogenic properties IL-8 could contribute to the development of new vessels that are found at the margins of tuberculous cavities during the healing process [13]. Moreover, Friedland et al. [14] have demonstrated that the inability to stimulate production of IL-8* ex vivo* correlated with poor prognosis in patients with TB.

It is known that several cytokines and growth factors have the ability to directly bind to bacterial molecules [15–22]. Therefore, we hypothesized that IL-8 could interact with* M. tb *and modulate the proinflammatory properties of this pathogen, especially since high concentrations of IL-8 are typically detected in patients with active TB [9, 10].

## 2. Methods

### 2.1. Human Subjects

All studies involving human blood and bronchoalveolar lavage fluids were approved by Human Subjects Investigation Committees of University of Tyler Health Science Center and University of Cincinnati Medical Center. Informed consent was obtained from both healthy volunteers and patients. The diagnosis of TB was confirmed by positive culture or using nucleic acid amplification testing (PCR). The diagnosis of MAC lung disease was based on criteria published by the American Thoracic Society/Infectious Diseases Society of America which have symptomatic, radiographic, and microbiologic components, the latter being the most important [23].

### 2.2. Purification of Monocytes and T Lymphocytes

White blood cells containing monocytes and lymphocytes were prepared from blood of normal donors by centrifugation through Ficoll Paque Plus (Pharmacia, Piscataway, NJ). Monocytes were then separated from other cells, mainly lymphocytes, by adherence to plastic. The adherent cells (monocytes) were incubated overnight with heat-killed* M. tb* (ATCC, Rockville, MD) to induce cytokine production. Then, conditioned media were collected and stored for further analysis. T lymphocytes were obtained by incubation of nonadherent cells with magnetic beads conjugated to CD3 (Dynal, Lake Success, NY). A magnetic cell separator was used to positively select CD3^+^ cells. CD4^+^ and CD8^+^ T lymphocytes were purified in a similar manner. The purity of the cells was tested by cytofluorometric analysis.

### 2.3. Chemotaxis

Chemotaxis of T lymphocytes was performed using Boyden chambers. Each chamber consisted of two compartments separated by a membrane. The lower compartment was filled with the stimulant, including conditioned media from* M. tb* stimulated monocytes and purified IL-8 alone (positive control). Then the membrane (a five-micron pore size polycarbonate filter, Nucleopore, Pleasanton, CA) was placed on the surface and the chamber was assembled. A 200 *μ*L aliquot of the T cell preparation was added to the top of the filter (the top compartment), and the chambers were incubated at 37°C for 4 h. After the incubation, the chambers were centrifuged for 20 min at 1,200 rpm to achieve settling of T lymphocytes, which migrated through the filters, at the bottom of the lower chambers. Then, the filter was removed, and the cells were counted using a hemocytometer (according to the manufacturer's instructions). In some experiments conditioned media from stimulated monocytes were incubated overnight with an antibody against IL-8 (R&D Systems, Minneapolis, MN), or monocyte chemotactic protein-1 (MCP-1) (R&D Systems, Minneapolis, MN), or macrophage inflammatory protein-1*α* (MIP-1*α*) (Serotec Inc., Raleigh, NC), or control antibody (mouse IgG1; Sigma, Chemical Co., St. Louis, MO) prior to performing the chemotactic assay.

### 2.4. Neutrophil Infection and Viability Determination

Blood was drawn from healthy volunteers, and neutrophils were purified according to the protocol routinely used in our laboratory [24]. Neutrophils were maintained in RPMI-1640 media supplemented with 2 mM L-glutamic acid, 1 mM sodium pyruvate, and 10% fetal bovine serum (Sigma, St. Louis, MO) at 37°C and 5% CO_2_. Cells were infected with* M. tbH37Rv* (ATCC, Rockwille, MD) at MOI of 1 : 10 for 30 min. Unbound bacteria were washed off and the cells incubated for an additional 3 h. After 3 h, infected neutrophils were lysed with 1 mL of 0.1% sodium dodecyl sulfate (SDS) in PBS. Appropriate dilutions of cell lysates were plated onto Middlebrook 7H10 agar supplemented with 10% Oleic Albumin Dextrose Catalase (OADC) enrichment. After 21 days of culture, the number of colony forming units (CFU) was counted. Killing index was calculated as percent of CFU at 30 min according to the following formula: ([CFU at 30 min − CFU at 3 hr] × 100/CFU at 30 min). In some experiments,* M. tbH37Rv* were incubated with IL-8 (final concentration 100 ng/mL per 5 × 10^7^ molecules of* M. tb*) overnight at 4°C, washed with PBS, and then used for infection.

### 2.5. Macrophage Infection and Viability Determination

Human monocytic leukemia cell line THP-1 (ATCC, Manassas, VA) was maintained in RPMI-1640 media supplemented with 2 mM L-glutamic acid, 1 mM sodium pyruvate, and 10% fetal bovine serum (Sigma, St. Louis, MO) at 37°C and 5% CO_2_. Prior to infection, THP-1 cells were exposed to 20 ng/mL phorbol-12-myristate-13-acetate (PMA) for 48 h to differentiate into macrophages (cells stop dividing and become adherent). THP-1 derived macrophages were infected with* M. tbH37Rv* (ATCC, Rockwille, MD) at MOI of 1 : 10 for 30 min. Unbound bacteria were washed off and the cells incubated for an additional 3 h. After 3 h, infected macrophages were lysed with 1 mL of 0.1% sodium dodecyl sulfate (SDS) in PBS. Appropriate dilutions of cell lysates were plated onto Middlebrook 7H10 agar supplemented with 10% Oleic Albumin Dextrose Catalase (OADC) enrichment. After 21 days of culture, the number of colony forming units (CFU) was counted. Killing index was calculated as percent of CFU at 30 min according to the following formula: ([CFU at 30 min − CFU at 3 hr] × 100/CFU at 30 min). In some experiments,* M. tbH37Rv* were incubated with IL-8 (final concentration 100 ng/mL per 5 × 10^7^ molecules of* M. tb*) overnight at 4°C, washed with PBS, and then used for infection.

### 2.6. Respiratory Burst (Fluorescence Assay)

Neutrophils mounted on microscope slides and THP-1 derived macrophages growing on coverslips were incubated with anti-phospho-p40phox antibody (Sigma) followed by Alexa 568 conjugated secondary antibody. Stained cells were analyzed using a Nikon Eclipse TE2000-U inverted microscope with a UV filter set. Intensity scan was created using an Ultraview Program (Perkin Elmer, Waltham, MA).

### 2.7. Measurement of IL-8

IL-8 concentrations were measured in an ELISA assay using matched antibody pair (R&D Systems, Minneapolis, MN).

### 2.8. Western Blot

Western Blot was performed to detect binding of IL-8 to mycobacterial proteins. Bacterial extracts were loaded into a 4–15% gradient SDS-PAGE gel. After electrophoresis the gel was subjected to electrophoretic transfer to a nitrocellulose membrane. The membrane was then blocked and incubated with IL-8. Next, anti-IL-8 antibody was applied and followed by enhanced chemiluminescence (ECL) reagents (Biosource, Camarillo, CA). The membrane was then exposed to X-ray film (Fuji Super RX).

### 2.9. Interaction of IL-8 with* M. tb* Molecules

*M. tb* growing in Middlebrook 7H9 broth supplemented with OADC and 0.05% Tween 80 were incubated with IL-8 (100 ng/mL final concentration) overnight at 4°C. The presence of IL-8 attached to* M. tb* molecules was visualized using anti-IL-8 antibody followed by Alexa 568 conjugated secondary antibody. Heated killed* M. tb* molecules and bacteria not incubated with IL-8 served as controls. Fluorescence was analyzed using a Nikon Eclipse TE2000-U inverted microscope with a UV filter set.

### 2.10. Statistical Analysis

Differences between groups were analyzed by a simple one way analysis of variance (ANOVA), or if the data were not normally distributed by a Kruskal-Wallis ANOVA on ranks. The direct comparison between any two treatment groups was performed using Student's *t*-test, or the nonparametric Mann-Whitney test when the data sets were not normally distributed. A *P* value of 0.05 or less was considered significant. All statistics were performed using SIGMA STAT (SPSS Science Inc., Chicago, IL).

## 3. Results

### 3.1. Human IL-8 Interacts with Tubercle Bacilli

**Figure 1:**
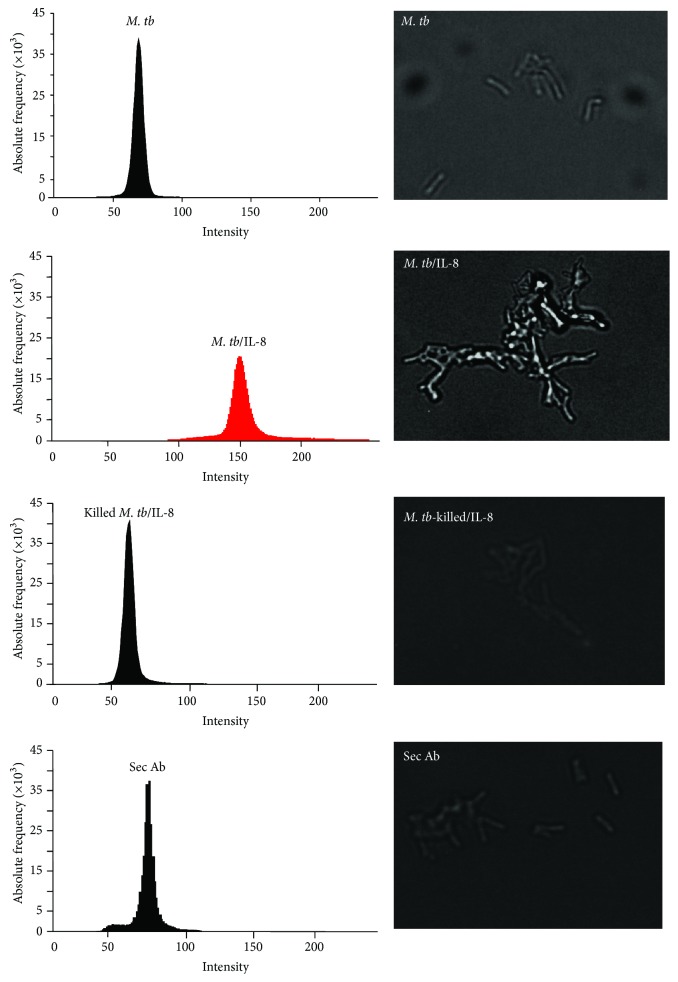
Binding of IL-8 to* M. tb*. Detection of IL-8 associated with* M. tb* using fluorescence microscopy.

It has been previously reported that the concentration of IL-8 increases in patients with active tuberculosis [9, 10, 25]. Kurashima et al. [9], for example, showed that the level of IL-8 in BAL fluid from TB patients was as high as 559.7 pg/mg albumin. Moreover, some bacterial pathogens can associate with proinflammatory factors [15–22]. Based on these facts we hypothesized that IL-8 could interact with tubercle bacilli. We first incubated IL-8 with the bacilli as described in Section 2 and used fluorescent microscopy to detect IL-8 bound to* M. tb* molecules. IL-8 associated with bacteria was visualized with a specific anti-IL-8 antibody followed by a secondary antibody conjugated with Alexa 568.* M. tb* cells cultured without IL-8 served as a negative control (Figure 1, top histogram). Tubercle bacilli coated with IL-8 and incubated only with the secondary antibody were used to demonstrate the specificity of anti-IL-8 antibody (Figure 1, bottom histogram). Microscope analysis revealed the presence of IL-8 attached to* M. tb* molecules (Figure 1, red histogram). We also used thermally killed tubercle bacilli in which the protein components were denatured to determine whether IL-8 binds to a surface protein of* M. tb*. We found that heat killed bacteria bound no IL-8 (Figure 1, second histogram from the bottom). This observation suggests that the mycobacterial “IL-8 receptor” may be a protein.

**Figure 2:**
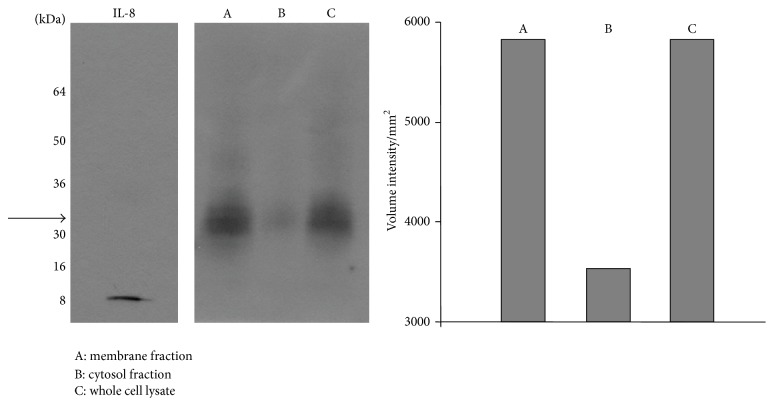
Binding of IL-8 to* M. tb*. Detection of IL-8 associated with cellular fractions of* M. tb* using Western Blot. The vertical bar chart depicts densitometric analysis of protein bands with Quantity One 1D Analysis Software (Bio-Rad).

To further confirm the ability of IL-8 to be associated with* M. tb* molecules, mycobacterial cell fractions were separated on a SDS PAGE gel. We noted that IL-8 bound to the membrane and whole cell lysate fractions but not to the cytosolic fraction (Figure 2). Detection of free IL-8 (Figure 2, left panel) served as a control of specificity of the anti-IL-8 antibody.

### 3.2. The Direct Association of IL-8 with* M. tb *Cells Leads to the Enhancement of the Ability of Inflammatory Cells to Phagocytose and Kill* M. tb*

Neutrophils are among the first cells attracted to a site of inflammation and play a critical role in the restricting of bacterial spread and in controlling the initial replication of bacteria [26]. Further, neutrophils which accumulate in the area of infection possess antimycobacterial qualities. These include the ability to phagocytize bacteria and elicit the oxidative burst which results in the production of reactive oxygen intermediates and degranulation, leading to the release of potent antimicrobial enzymes [12, 27, 28].

**Figure 3:**
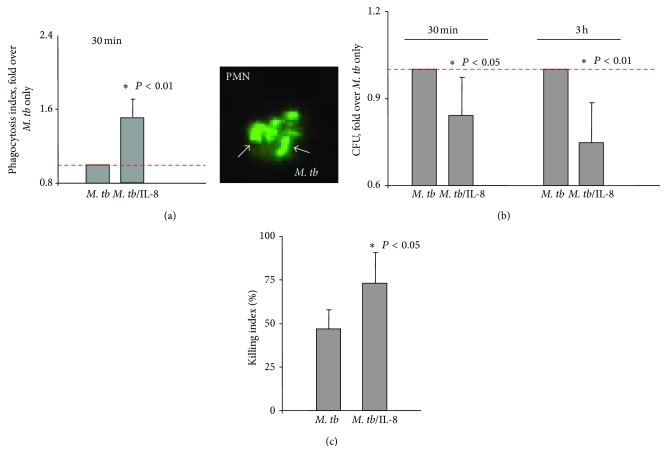
Effect of direct association of IL-8 with* M. tb *molecules on the ability of neutrophils to phagocytose and kill* M. tb*. (a) Phagocytosis of* M. tb* only (*M. tb*), or* M. tb* bound to IL-8 (*M. tb*/IL-8) presented as a phagocytosis index/increase fold over* M. tb* only. Image of neutrophil phagocyting* M. tb* conjugated with FITC photographed under fluorescent microscope. (b) Phagocytosis and killing of* M. tb* only (*M. tb*), or* M. tb* associated with IL-8 (*M. tb*/IL-8) calculated after 30 min and 3 h of infection using colony forming units (CFU) and presented as a CFU/fold over* M. tb* only. (c) Killing index (%) calculated for* M. tb* cytokine-free (*M. tb*) and* M. tb* associated with IL-8 (*M. tb*/IL-8) according to the formula ([CFU at 30 min − CFU at 3 h] × 100/CFU at 30 min).

We tested proinflammatory functions of* M. tb* molecules coated with IL-8 using purified neutrophils. Neutrophils purified from blood of healthy volunteers [24] were infected with tubercle bacilli coated with IL-8 (100 ng of IL-8 per 5 × 10^7^ bacteria) or not coated. An index of phagocytosis was determined after 30 min of infection using fluorescent microscopy (*M. tb* conjugated with fluorescein—FITC). At least 100 neutrophils were evaluated. We also assessed phagocytosis by counting colony forming units (CFU). We found that phagocytic uptake of IL-8/*M. tb* complexes by neutrophils was substantially enhanced (*P* < 0.01). Accordingly, the phagocytic index (number of ingested bacteria per cell) was 5.0 for IL-8-coated bacteria (*M. tb/*IL-8) and 3.0 for control bacteria (*M. tb*). Therefore, the association of IL-8 with* M. tb* triggered a substantial increase (approximately 1.5 times) in the ability of neutrophils to phagocyte bacteria (Figure 3(a)). Furthermore, analysis of 3 h infection indicated that the presence of IL-8 downregulated the infectious potential of* M. tb*. As shown in Figure 3(b), neutrophils killed bacilli opsonized with IL-8 more effectively than IL-8-free bacteria. Analysis of the CFU showed that the total number of* M. tb* was lower at both time points (30 min and 3 h) for bacteria preincubated with IL-8 in comparison to the number of IL-8-free* M. tb* (*P* < 0.05 and < 0.01, resp.) This observation was further confirmed by calculating killing index as shown in Figure 3(c) (*P* < 0.05).

**Figure 4:**
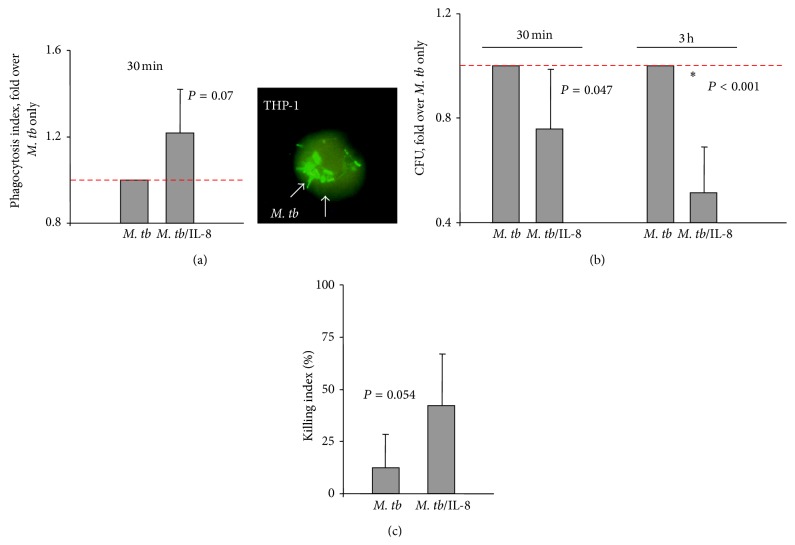
Effect of direct association of IL-8 with* M. tb *molecules on the ability of THP-1 cells to phagocytose and kill* M. tb*. (a) Phagocytosis of* M. tb* only (*M. tb*), or* M. tb* bound to IL-8 (*M. tb*/IL-8) presented as a phagocytosis index/increase fold over* M. tb* only. Image of THP-1 cell phagocyting* M. tb* conjugated with FITC photographed under fluorescent microscope. (b) Phagocytosis and killing of* M. tb* only (*M. tb*), or* M. tb* associated with IL-8 (*M. tb*/IL-8) calculated after 30 min and 3 h of infection using colony forming units (CFU) method and presented as a CFU/fold over* M. tb* only. (c) Killing index (%) calculated for* M. tb* cytokine-free (*M. tb*) and* M. tb* associated with IL-8 (*M. tb*/IL-8) according to the formula ([CFU at 30 min − CFU at 3 h] × 100/CFU at 30 min).

Macrophages are the second major group of inflammatory cells involved in the innate immunity response against microorganisms. They are implicated in phagocytosing and killing mycobacteria and are considered the primary host cells for mycobacteria [29]. THP-1 cells have become one of the most widely used cell lines to investigate biological functions of monocytes and macrophages as they relate to various diseases, including TB [30]. In our studies, THP-1 cells were differentiated into macrophages with PMA and then infected with* M. tb* coated with IL-8 (100 ng of IL-8 per 5 × 10^7^ bacteria) or uncoated bacteria. Analysis of phagocytic index showed that the association of IL-8 with* M. tb* enhanced the ability of macrophages to ingest bacteria 1.22 times but this difference did not reach statistical significance (*P* = 0.07; Figure 4(a)). The analysis of the CFU at 3 h indicated that the total number of* M. tb*/IL-8 was substantially (*P* < 0.001) decreased in comparison to the number of IL-8-free* M. tb* bacilli (Figure 4(b)). However, the killing index did not differ for these 2 groups as shown in Figure 4(c) (*P* = 0.054).

**Figure 5:**
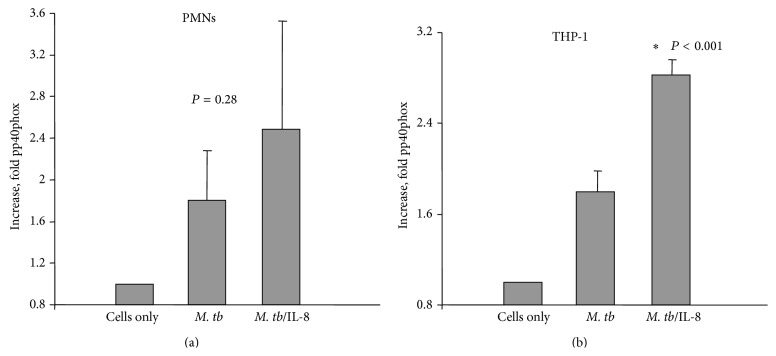
Respiratory burst presented as a level of pp40phox component detected using fluorescent microscopy. (a) Level of pp40phox molecule in neutrophils infected with* M. tb* cytokine-free (*M. tb*) or* M. tb* associated with IL-8 (*M. tb*/IL-8) presented as an intensity scan/increase fold over normal cells. (b) Level of pp40phox molecule in THP-1 cells infected with* M. tb* cytokine-free (*M. tb*) or* M. tb* associated with IL-8 (*M. tb*/IL-8) presented as an intensity scan/increase fold over normal cells.

The protein p40phox is a major constituent of NADPH-oxidase, which is a multicomponent enzyme system responsible for the oxidative burst [31, 32]. As a consequence of stimulation of inflammatory cells p40phox is phosphorylated and translocates from the cytosol to the cell membrane to form an enzymatic complex which produces oxygen radicals. Analysis of the level of phospho-p40phox in neutrophils and THP-1 derived macrophages infected with* M. tb* was performed using fluorescent microscopy. As shown in Figure 5(a), the presence of IL-8 associated with* M. tb* molecules caused the increase (approximately 1.38 times) in the level of pp40phox in neutrophils compared to chemokine-free bacteria but the difference was not statistically significant (*P* = 0.28). Similar observations were made using THP-1 derived macrophages stimulated with IL-8 coated or uncoated* M. tb* cells (Figure 5(b)). Analysis of fluorescence intensity showed that cells infected with* M. tb* opsonized with IL-8 appeared to be more potent (1.57 times) producers of phospho-p40phox (*P* < 0.001; Figure 5(b)).

### 3.3. IL-8 Concentration in Blood and Lung Fluids from TB Patients: Clinical Aspect of IL-8 in Tuberculosis

IL-8 concentrations have been measured by several investigators in both plasma and BAL fluids from TB patients. However, the results are quite variable [9, 10, 33–35]. Therefore we evaluated concentrations of this chemokine in blood and BAL fluids from patients with TB and for comparison in samples from patients with mycobacteriosis caused by* Mycobacterium avium-complex* (MAC). It has to be noted that IL-8 produced by white blood cells is present in blood in two forms: soluble (plasma IL-8) and cell-associated (IL-8 associated with red blood cells). It has been shown that a significant percentage of IL-8 in blood is associated with red blood cells. Erythrocytes express a receptor that binds multiple chemokines, including IL-8 [36, 37]. We measured both types of IL-8 in blood from 7 healthy volunteers, 14 TB patients, and 13 patients infected with MAC. We showed for the first time that levels of IL-8 associated with red blood cells from TB patients, MAC patients, and healthy donors were significantly different (*P* < 0.05) (Table 1). In agreement with previous reports [24] we found that IL-8 concentrations were significantly higher in plasma from TB patients than in plasma from normal subjects (*P* < 0.02) (Table 2). Our data also demonstrated (Table 2) that amounts of IL-8 were substantially elevated in plasma from patients with MAC compared to the amounts of this chemokine in plasma from normal subjects. Moreover, we evaluated concentrations of IL-8 in BAL fluids from TB patients, patients with MAC, and healthy subjects (Table 3). The levels of IL-8 were significantly higher (*P* < 0.001) in the two patient groups. Previous reports also demonstrated that lung fluids from TB patients contained significant amounts of IL-8 [9, 10]. We also noted that concentrations of IL-8 were lower in plasma than in bronchoalveolar lung lavage fluids.

### 3.4. IL-8 Triggers Chemotaxis of T Lymphocytes

**Figure 6:**
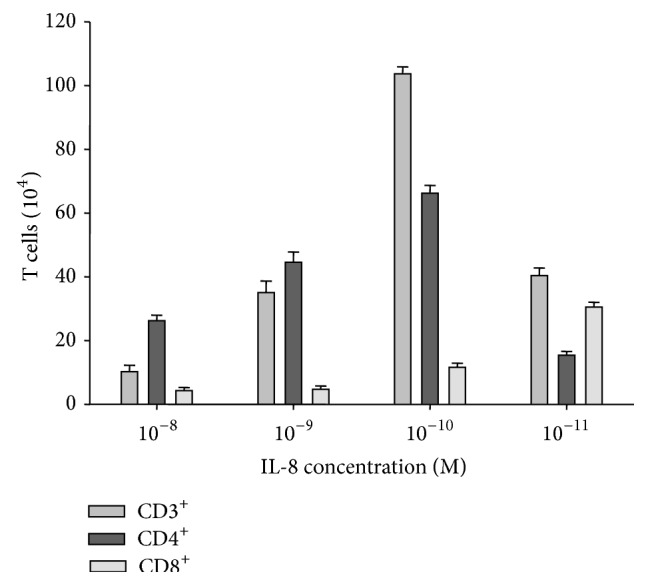
IL-8-induced chemotaxis of T lymphocytes.

Since the role of IL-8 as a chemoattractant for T lymphocytes remains controversial [38–41], we evaluated the ability of IL-8 to recruit these cells. We observed that IL-8 induced a significant chemotaxis of T lymphocytes (Figure 6). This response was highly reproducible and dose dependent (Figure 6). Moreover, both CD4^+^ and CD8^+^ T cell subsets play a central role in developing resistance to* M. tb* [42]; thus, CD4^+^ and CD8^+^ T lymphocytes were also tested in the chemotactic assay (Figure 6). These cells were isolated using magnetic beads conjugated to CD4 or CD8 (purity of >95%). We found that both CD4^+^ and CD3^+^ T cells reached maximum response at an IL-8 concentration of 10^−10^ M (Figure 6). The response of CD8^+^ T lymphocytes was weaker than of other cell types and reached a maximum at an IL-8 concentration of 10^−11^ M (Figure 6).

**Figure 7:**
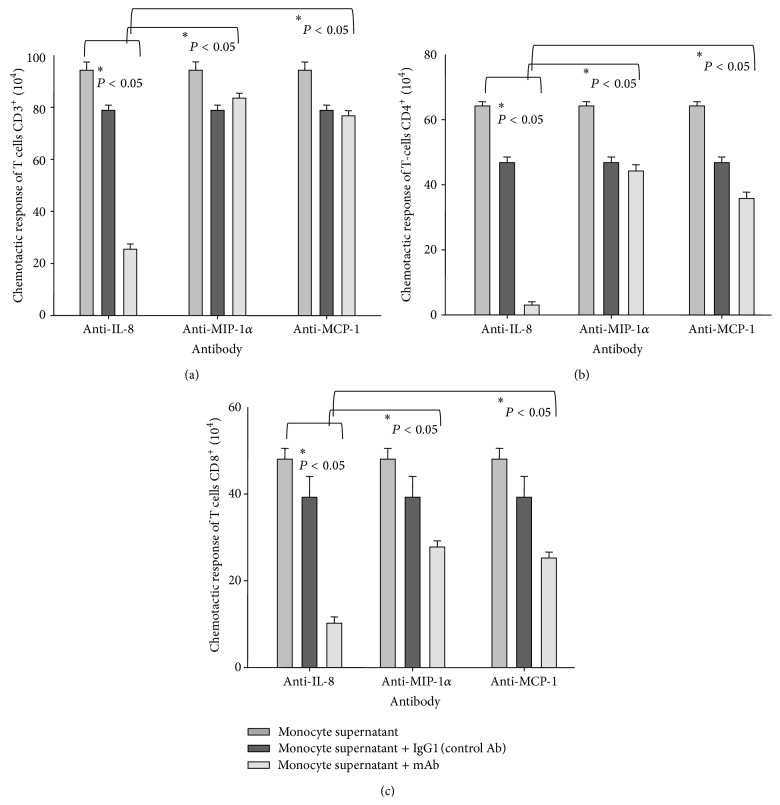
Chemotaxis of CD3^+^ (a), CD4^+^ (b), and CD8^+^ (c) cells from a healthy subject triggered by conditioned media from* M. tb* stimulated monocytes. Effect of anti-IL-8, anti-MIP-1*α*, and anti-MCP-1 antibodies.

TB is considered a granulomatous disease due to the accumulation of significant numbers of neutrophils, monocytes, and T lymphocytes in the infected area [2]. However, specific chemokines responsible for recruiting these cells have not yet been identified. We used conditioned media from human monocytes stimulated with heat-killed* M. tb* to evaluate the contribution of IL-8 to chemotactic responses of T lymphocytes. We noted that monocytes stimulated with* M. tb* produced approximately 100 ng/mL of IL-8. Conditioned media induced a significant chemotaxis of CD3^+^, CD4^+^, and CD8^+^ T cells. Furthermore, we found that the chemotactic response of T lymphocytes was substantially inhibited by a monoclonal anti-IL-8 antibody (*P* < 0.05) and not by the control antibody. To examine the possibility that other chemokines, which are produced by the stimulated monocytes, contribute to the migratory activity of the conditioned media, we tested specific neutralizing antibodies against chemokines with known activity towards T cells. These include macrophage inflammatory protein-1*α* (MIP-1*α*) and macrophage chemotactic protein-1 (MCP-1). Our results indicated that anti-MIP-1 *α* antibody or anti-MCP-1 antibody is less effective (*P* < 0.05) than anti-IL-8 antibody in suppressing the chemotactic response of CD3^+^ (Figure 7(a)) as well as CD4^+^ and CD8^+^ T lymphocytes (Figures 7(b) and 7(c)).

## 4. Discussion

The function of IL-8 in the pathogenesis of TB is controversial and a major goal in the following studies was to investigate the involvement of IL-8 in modulating the immune and inflammatory responses during infection with* M. tb*. There are several studies showing that some growth factors, cytokines, or other mediators have the ability to interact with bacterial pathogens affecting the course of inflammation [15–22]. C3-binding molecules, for example, were detected on the surface of several intracellular pathogens, including* Legionella pneumophila*,* Chlamydia trachomatis*,* Leishmania*, and* Mycobacterium leprae* [18–20]. Bermudez et al. [21, 22] studied epidermal growth factor-binding protein in* Mycobacterium avium* and* M. tb*. In addition, Luo et al. [16] investigated TNF-*α* binding to* Shigella flexneri*. We analyzed the interaction between IL-8 and tubercule bacilli and showed for the first time that IL-8 could directly associate with* M. tb*. We also found that as a consequence of this interaction antimicrobial activities of neutrophils and macrophages were enhanced. Moreover, the amount of IL-8 that was used to coat the bacteria appears to be physiologically relevant (discussed in the next paragraph).

There is some information in the literature concerning the concentration of IL-8 in plasma and BAL fluids of patients with active TB [9, 10, 25, 33–35]. Sadek et al. [10] showed that levels of IL-8 were increased in BAL fluid by 8.9-fold, whereas we found that IL-8 concentrations were increased 91 times in BAL fluids of patient with TB in comparison to healthy subjects (Table 3). It is important to mention that the increase of IL-8 concentration would be even more significant considering the fact that the BAL method dilutes alveolar fluids by 50- to 100-fold [43].

In agreement with previous findings [44] we have shown that the concentration of IL-8 in plasma and BAL fluids from patients with MAC disease was significantly increased in comparison to normal subjects. It should be noted that we are the first group to show that high concentration of IL-8 associates with red blood cells in TB patients and subjects with MAC disease.

There are a limited number of studies reporting the role of IL-8 as a chemoattractant for T cells [38–41]. Xu et al. [38] showed that IL-8 was as potent as RANTES, MIP-1*α*, and MIP-1*β* in inducing chemotaxis of CD3^+^ T lymphocytes. They tested several concentrations of IL-8 and found that the optimal concentration of IL-8 ranged from 10 to 50 ng/mL (1–6 × 10^−9^ M) [38]. We demonstrated that IL-8 present in culture media of monocytes stimulated with heat-killed* M. tb* was significantly more effective in inducing CD3^+^ T cell chemotaxis than MIP-1*α* and MCP-1 (Figure 7). This is in contrast to Sadek et al. [10], who reported similar activity for IL-8, RANTES, MCP-1, and MIP-1*α*. Furthermore, we observed that the chemotactic migration of CD3^+^ T cells was concentration dependent and reached a maximum response at an IL-8 concentration of 10^−10^ M (Figure 6).

Zachariae et al. [39] questioned the ability of IL-8 to attract CD4^+^ and CD8^+^ T lymphocytes equally well. In addition, the authors stated that IL-8 at concentration of 100 ng/mL (1.2 × 10^−8^ M) attracted CD8^+^ T lymphocytes more efficiently. Taub et al. [40] demonstrated that IL-8 was a potent T lymphocytes migratory agent* in vivo* in SCID mice. However the effect which IL-8 had on T lymphocytes chemotaxis* in vitro* was indirect. According to the authors [40] IL-8 induced neutrophil stimulation and degranulation which further caused T cells migration. The neutralization analysis showed that the majority of neutrophil granule-induced T cell migration was not due to chemokines [40]. We found that IL-8 was a potent attractant for CD4^+^ and CD8^+^ T lymphocytes and that migration of these cells towards IL-8 reached a maximum at concentrations of 10^−10^ M and 10^−11^ M for CD4^+^ and CD8^+^ T cells, respectively (Figure 6).

In summary, chemokines, including IL-8, are produced in lungs in response to* M. tb* infection. They induce immune cell recruitment to the lungs and play an important role in granuloma formation. They also act as key regulators of host defense against* M. tb* infection. Our novel observations indicate that IL-8 has the ability to directly interact with* M. tb* and in this way enhance antimicrobial functions of proinflammatory cells, that is, macrophages and neutrophils.

## Figures and Tables

**Table 1:** Concentration of IL-8 (pg/mL) associated with red blood cells in human blood from: normal subjects (7 subjects), patients infected with MAC (13 patients), and patients infected with TB (14 patients).

IL-8 associated with red blood cells (pg/mL)		
Normal	MAC	TB
53.8 ± 17.6	103.8 ± 82.5^*^	125.3 ± 91.3^**^

Values are means ± SD.

^*^ *P* < 0.05 compared with normal.

^**^ *P* < 0.05 compared with normal.

**Table 2:** Concentration of IL-8 (pg/mL) in human samples; **A/**IL-8 in human plasma from: normal subjects (7 subjects), patients infected with *Mycobacterium Avium Complex *(MAC, 13 patients), and patients infected with TB (14 patients).

IL-8 in plasma (pg/mL)		
Normal	MAC	TB
20.7 ± 26.2	64.0 ± 85.4^*^	64.1 ± 77.6^**^

Values are means ± SD.

^*^ *P* < 0.02 compared with normal.

^**^ *P* < 0.02 compared with normal.

**Table 3:** Concentration of IL-8 (pg/mL) in lung fluids from: normal subjects (10 subjects), patients infected with MAC (10 patients), and patients infected with TB (15 patients).

IL-8 in lung fluids (pg/mL)		
Normal	MAC	TB
4.0 ± 4.4	141.3 ± 334.6^*^	365.6 ± 794.9^**^

Values are means ± SD.

^*^ *P* < 0.001 compared with normal.

^**^ *P* < 0.001 compared with normal.
